# Spontaneous Vertebral Artery Dissection: A Commonly Overlooked Cause of Headache

**DOI:** 10.7759/cureus.80962

**Published:** 2025-03-21

**Authors:** Catherine A Ostos Perez, Kristina Menchaca, Erika Ostos Perez, Shaun Isaac

**Affiliations:** 1 Internal Medicine, University of Miami John F. Kennedy (JFK) Medical Center, Atlantis, USA; 2 Biological Sciences, St. Petersburg College, Clearwater, USA

**Keywords:** anticoagulation, arterial dissection, bleeding risk, cephalea, cervical arterial dissection, headache disorders, posterior circulation stroke, stroke, vertebral artery, vertebral artery dissection

## Abstract

The differential diagnosis of headaches is very diverse, and they can represent the symptoms of life-threatening conditions that need to be addressed promptly. Vertebral artery dissection (VAD) can be manifested with isolated headaches, and it can be easily overlooked. It usually presents in younger patients along with some focal neurological symptoms. It is diagnosed with multiple imaging modalities.

We present a case of a 38-year-old woman without evident risk factors and an isolated headache who was only diagnosed with a spontaneous VAD after multiple primary care office visits and even an urgent care facility before presenting to the Emergency Room. We will review the characteristics of the pain related to VAD to improve its identification in the outpatient setting. This is a potential area of research and quality improvement to diagnose and treat this condition promptly.

## Introduction

The International Classification of Headache Diseases - Third edition (ICHD-3) divides headaches into primary and secondary headaches, painful cranial neuropathies, other facial pain, and other headaches [1]. It is one of the most common symptoms in outpatient and inpatient settings and it is important to determine the etiology to rule out potentially life-threatening conditions such as stroke, vasculopathy, infections, and brain tumors among others. Headaches also can often be the manifestation of systemic disease [2]. Computerized tomography (CT) or magnetic resonance imaging (MRI) of the brain, transcranial Doppler (TCD), digital subtraction angiography (DSA), lumbar puncture (LP), and genetic testing may be needed to identify the potential life-threatening etiologies [1].

Although one of the most common presentations of vertebral artery dissection (VAD) is headache, it is a rare cause of isolated headaches [1-6]. Moreover, VAD can be caused by a recent trauma or cervical manipulation. VAD is a life-threatening known cause of stroke in young adults [1,3,5,6]; however, in the absence of neurological symptoms, it can be overlooked. We present a case of a young woman without evident risk factors, who was diagnosed with a spontaneous VAD with its only manifestation being an isolated headache.

## Case presentation

A 38-year-old female with no relevant past medical history or comorbid conditions presented to the emergency department with a three-week onset of progressively worsening headache and neck pain. The pain started in the left mastoid region and radiated up her head and down through her left neck with an intensity of 3/10 and progressive to an 8/10. The pain was described as constant, throbbing, and on occasion pulsatile, with no aggravating or alleviating factors. She denied visual impairment, no sensory or motor abnormalities, no dizziness, no nausea or vomiting, no lacrimation, no photophobia, no difficulty chewing, no dysarthria, no changes in consciousness, or any other neurological abnormalities were reported. She denied any recent history of trauma. The patient has experienced similar episodes because she stated she thought she might have slept in an uncomfortable position; therefore, she took non-steroidal-anti-inflammatories (NSAIDS) over the counter without improvement.

She visited within a span of three weeks, two different primary care offices and an urgent care facility where she was prescribed topiramate, NSAIDS, and opiates to relieve the pain, and was even referred to physical therapy but was unable to attend yet, and the pain was becoming incapacitating. No history of cervical manipulation was reported either. On the review of systems, she admitted palpitations; she denied fever, chills, respiratory, gastrointestinal, musculoskeletal, dermatological, or genitourinary symptoms.

On the physical exam vital signs with hypertension BP 180/92 mmHg, the rest was within normal ranges. The patient was alert, oriented in time, space, and person, clear, coherent, and fluent speaking, no cranial nerve abnormalities were noted, no dysarthria, dysmetria, no nystagmus. Strength and sensation were preserved in all her muscle groups, deep tendon reflexes were normal as well. She did not have any warmth, or skin rashes, and she did admit tenderness on her left neck upon palpation. The range of motion of the neck was limited due to pain.

**Figure 1:**
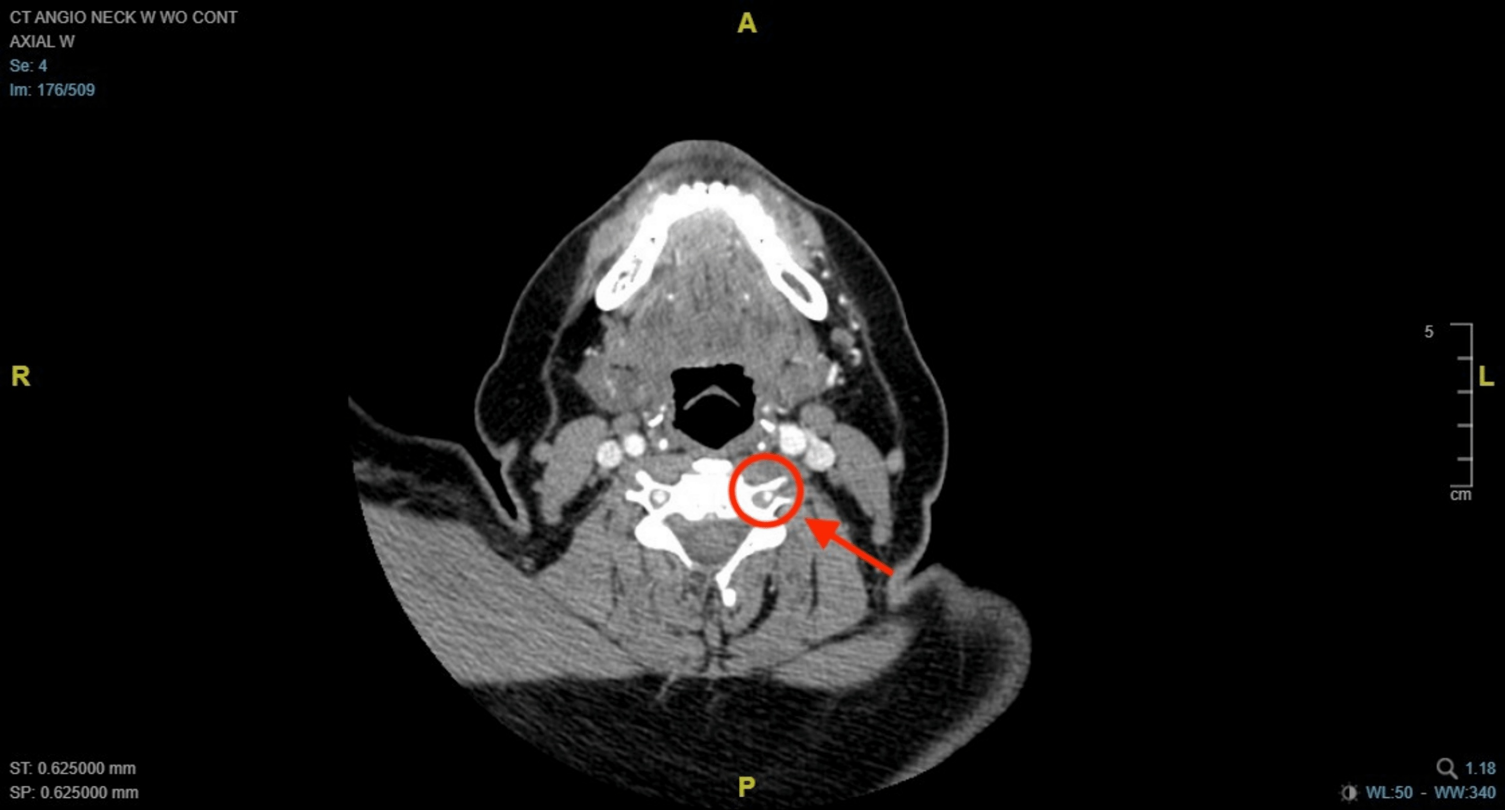
(Axial view) Computerized tomography angiography (CTA) of the neck with and without contrast showing peripheral hypoattenuation and luminal narrowing of the left vertebral artery, compared to the right side (red circle and arrow).

Initial laboratory work was unremarkable. CT angiography (CTA) was done which revealed peripheral hypoattenuation with luminal narrowing of the V3 segment left vertebral artery with irregularity and diminutive appearance of the proximal V4 segment. Appearance is concerning for VAD (Figure 1).

**Figure 2:**
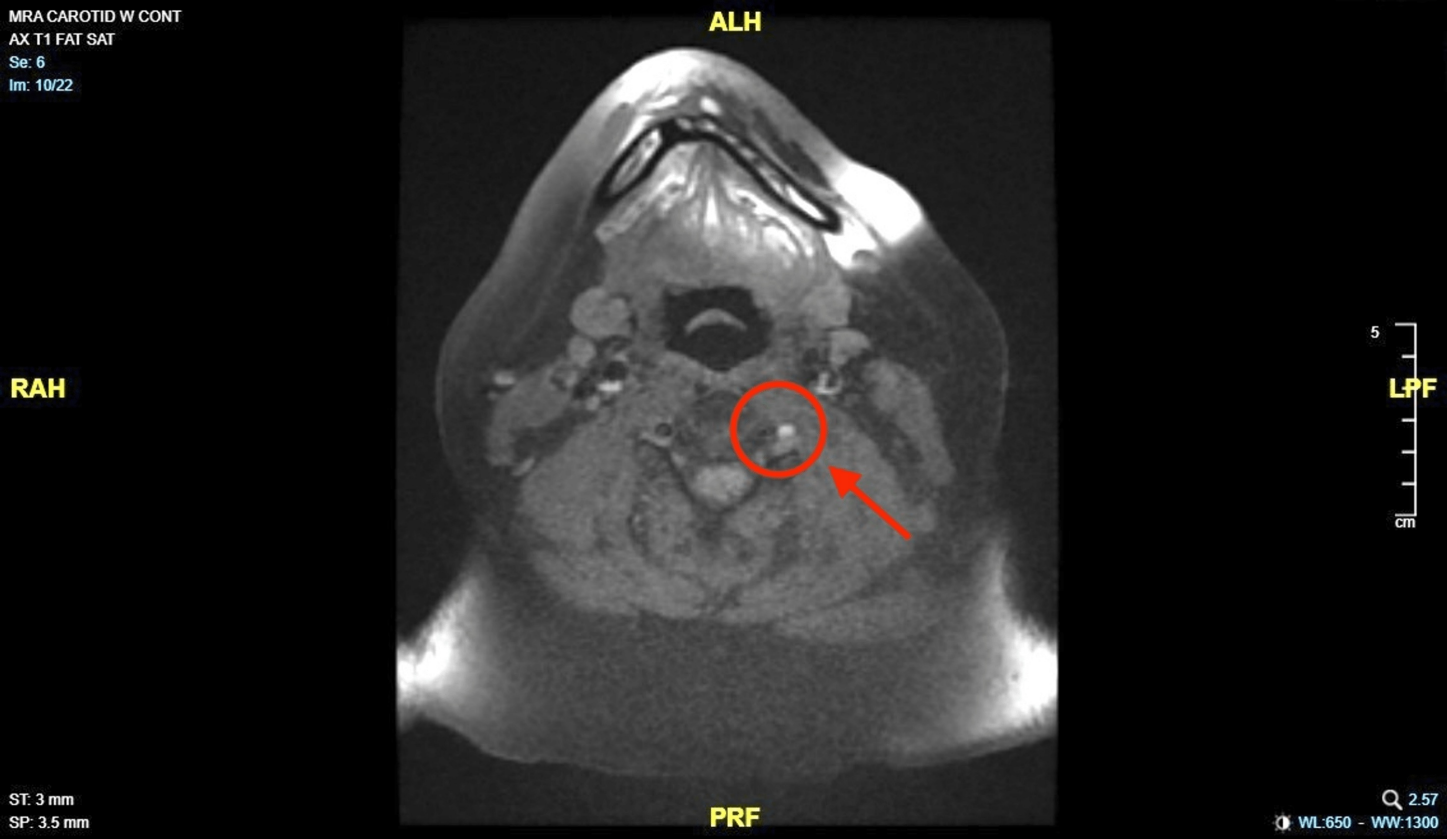
(Axial view) Magnetic resonance angiogram (MRA) of neck showing opacification of the left vertebral artery and T1 hyperintense signal, compared to the right side (red circle and arrow).

Further imaging with MRI and Angiogram (MRI/MRA) of the brain and neck revealed intermittent opacification of the left vertebral artery without changes compared to initial imaging. It had a thickened wall with some T1 hyperintense signal which may represent evidence of dissection. The V4 segment of the left vertebral artery appears to be severely stenotic. No central filling defect was seen within the middle, anterior, and posterior cerebral arteries (Figure 2).

During her seven-day hospital stay, she was assessed by neurology, cardiology, and vascular surgery. No surgical intervention was done due to time-of-onset. She was managed conservatively with intravenous heparin and transitioned to oral anticoagulation with warfarin; also started on aspirin and statin and was discharged when stable. The patient did not have any neurological sequelae.

## Discussion

Although a broad classification of headaches exists, there is a group of headaches encompassing those related to cranial and/or cervical vascular disorders such as cerebral ischemic events, non-traumatic intracranial hemorrhage, unruptured vascular malformation, arteritis, cervical carotid or vertebral artery disorder, and pituitary apoplexy [1]. This type of headache is usually of insidious onset, throbbing nature, although can be abrupt and may or may not associated with other local symptoms and signs.

Collecting a detailed medical history is extremely important, to identify the possible source of the headache, especially where it involves recent trauma, onset, duration, and associated neurological and non-neurological symptoms, and to decide if further workup with advanced imaging is necessary [1]. Many life-threatening conditions, such as ischemic or hemorrhagic-associated headaches are overshadowed by focal signs. Some cerebral vascular diseases can cause headache and stroke simultaneously, such as SAH, vasculitis, artery dissection, and cerebral venous thrombosis (CVT), in which headache is usually a warning symptom. Thus, it is essential to be aware of the relationship between headaches and the aforementioned diseases [1].

Cervical artery dissection is divided into internal carotid artery dissection and VAD, in which blood flows into the wall of the cervical artery to form an intramural hematoma, which can lead to intraluminal thrombus, embolization, and occlusion, or aneurysmal changes of the artery. The average age of onset is around 44 years. It represents about 2% of all ischemic strokes and 20%-25% of ischemic strokes in young adults [3,5-7]; it occurs spontaneously in about 60% of cases but can be related to trauma or mechanical triggers as well. The majority of patients present with neck pain or headache associated with neurological deficits due to ischemic stroke or local symptoms, even though neck pain and headache, alone or in combination, are the only symptoms in approximately 15%-20% of cases. Diagnosis may be missed especially when headache is the only symptom or when clear neurological deficits are absent [1,2]. In our case, this was the only presentation, and therefore the diagnosis was delayed by the patient visiting multiple facilities prior to the ER where she had imaging that led to the final identification of the cause of her headache.

The main characteristic of VAD is the development of an intramural hematoma [3]. The exact mechanisms that lead to this formation are unclear, although there are some hypotheses that suggest that it could be due to a subintimal tear, or intramural rupture of the vasa vasorum [3,5,7]. The pain is caused by stretching and irritation of the adventitia in the blood vessel wall which has considerable nociceptive innervation [5].

Headache associated with cervical artery dissection has no specific features and sometimes it mimics others such as migraine and clusters [1]. The pain usually resolves within a week in the majority of patients, but sometimes it can persist for many years [3]. If the patient only suffers from neck pain, the degree of dissection is often mild to moderate [1,3,7]. Neurological deficits due to ischemia may occur in up to 90% of cases of VAD and may involve the brain stem, thalamus, and cerebral or cerebellar hemispheres [3]. In our case, this is even more rare, since there were no ischemic neurological manifestations or focal signs on physical exam.

VAD should be seriously considered when dealing with patients complaining of the first attack of headache that mimics migraine or those with cervicogenic headaches, which fail to respond to the usual treatment. Moreover, posterior circulation stroke among young patients or stroke with pain in the head and neck should be investigated carefully with extensive neuroimaging [4]. The case we described warranted further imaging due to its progression and lack of improvement with pain medications.

Dissection can be identified by a variety of different imaging modalities including CTA, MRI/MRA, and DSA, and it can show long, irregular stenosis, an occlusion, intraluminal thrombus, or a dissecting aneurysm [5]. Pathognomonic features of dissection, such as an intimal flap or a double lumen, are detected in fewer than half of reported cases. They are dynamic processes, thus radiographic findings may change dramatically within a period of days or even hours [1,3].

Some heritable connective-tissue disorders are associated with an increased risk of spontaneous dissections of the carotid and vertebral arteries. Familial and genetic factors have been described as well [3]. Other risk factors for stroke from VAD include a precipitating event including hyperextension or rotation of the neck including practicing yoga, painting a ceiling, coughing, vomiting, sneezing, the receipt of anesthesia, and the act of resuscitation, chiropractic manipulation. These sudden neck movements may cause injury in the artery by mechanical stretching [3].

In general, patients receive intravenous heparin followed by warfarin for all patients with VAD, regardless of symptoms to prevent thromboembolic complications, unless there are contraindications such as intracranial extension of the dissection [3]. Anticoagulation is recommended for three to six months with an international normalized ratio (INR) goal of 2-3 [3]. Some associated risks for this treatment might be enlargement of mural hematoma, and perforation [5]. Patients with intracranial VAD have a higher risk of developing subarachnoid hemorrhage. In this circumstance, the use of APs and anticoagulants (ACs) should be cautioned [6]. Both AP agents and ACs are used to reduce stroke risk, but whether one or both treatment strategies are more effective is unknown [8].

Similar to other causes of stroke, which can be treated with procedures such as intravenous thrombolysis (IVT) and endovascular therapy (EVT), or thrombectomy, the stroke symptoms caused by VAD have a different pathophysiological mechanism, which is not cardioembolic or atherosclerosis, therefore the approach is more conservative [3].

The patient in this case was young, without known comorbidities with a subacute headache that can be easily overlooked in the outpatient setting given her lack of risk factors for a stroke and focal neurological signs that could have prompted imaging earlier on. The characteristics of the pain were different than the ones described in the literature; however, it is known that VAD pain can mimic innocuous headaches. The importance of monitoring patients with VAD is emphasized due to the high risk of developing a stroke after the dissection; therefore, our patient was followed up at the clinic and monitored her anticoagulation and APs.

## Conclusions

Headache is a common symptom in patients presenting to the outpatient and primary care settings, although a lot of them can be suggestive of something non-urgent, it is important to keep in mind dangerous etiologies. Cervical dissections including VAD are rare but dangerous lesions that can be hard to diagnose and can be overlooked in younger patients who otherwise appear healthy. Any patient with refractory, first-time pain, or progressive symptoms needs a thorough evaluation to determine the causes. Timely and accurate diagnosis of VAD with proper treatment is crucial for good outcomes and preventing disability.

More research on etiology, pathophysiology, and potential quality improvement projects can be developed regarding how often dangerous headaches occur to improve early detection and treatment to develop guidelines for specific management of patients with stroke secondary to VAD and the possible role of other endovascular procedures and complications of management with anticoagulation in the long term.
